# Efficacy of* Scutellaria baicalensis* for the Treatment of Hand, Foot, and Mouth Disease Associated with Encephalitis in Patients Infected with EV71: A Multicenter, Retrospective Analysis

**DOI:** 10.1155/2016/5697571

**Published:** 2016-10-20

**Authors:** Hailong Lin, Jian Zhou, Kaichun Lin, Hongjiao Wang, Zunhong Liang, Xingshuai Ren, Leting Huang, Chan Xia

**Affiliations:** ^1^Department of Pediatrics, The Second Affiliated Hospital of Wenzhou Medical University, Wenzhou, China; ^2^Department of Pediatrics, The First People's Hospital of Yongkang, Jinhua, China; ^3^Department of Pediatrics, The Affiliated Children's Hospital of Zhejiang University Medical College, Hangzhou, China; ^4^Department of Pediatrics, Hainan Provincial People's Hospital, Haikou, China; ^5^Department of Pediatrics, Zouping People's Hospital, Binzhou, Shandong Province, China

## Abstract

This study aimed to evaluate the clinical efficacy and safety of using the traditional Chinese herbal medicine* Scutellaria baicalensis* for the treatment of severe HFMD in 725 patients aged >1 year in a multicenter, retrospective analysis. The patients were divided into the* S. baicalensis* and ribavirin groups, and the temperatures, presence or absence of skin rashes and oral lesions, nervous system (NS) involvement, and viral loads of the patients, as well as the safety of the treatments, were evaluated. The median duration of fever, median time to NS involvement, and the number of patients with oral ulcers and/or vesicles, as well as skin rashes, were decreased in the* S. baicalensis* group compared with the ribavirin group. In addition, the EV71 viral loads were decreased in the* S. baicalensis* group, suggesting that* S. baicalensis* exerted more potent antiviral effects compared with ribavirin. The present study demonstrated that* S. baicalensis* was suitable for the treatment of severe HFMD in patients aged >1 year, since it was shown to rapidly relieve fever, attenuate oral lesions and rashes, and improve NS involvement. Furthermore, it was demonstrated to be relatively safe for topical application.

## 1. Introduction

Hand, foot, and mouth disease (HFMD) is a common infectious disease caused by enteroviruses and predominantly occurs in infants and children aged <7 years [1]. Although the majority of cases are mild, some develop into severe cases. Severe HFMD, which is usually caused by enterovirus 71 (EV71) infections, is characterized by the complication of encephalitis, which may even develop into brainstem encephalitis or neurogenic pulmonary edema [2, 3]. The treatment of severe HFMD is challenging owing to the lack of effective antiviral drugs. Ribavirin has been shown to have no significant effect, while other antiviral drugs have not been recommended for the treatment of HFMD [4]. Some traditional medication extracts have demonstrated antiviral effects, including the traditional Chinese herbal medicine* Scutellaria baicalensis *[5], which was the only herbal medicine to be recommended for the treatment of HFMD in 2012 by the Ministry of Health, China. Although single center articles investigating the efficacy of* S. baicalensis* have been published in Chinese language journals, no previous study has performed a multicenter analysis. Therefore, the present study collated data from five hospitals distributed across China in order to retrospectively analyze the efficacy of* S. baicalensis*, as compared with ribavirin, for the treatment of HFMD with concomitant encephalitis.

## 2. Methods

The present study retrospectively analyzed the medical records of hospitalized patients with HFMD and encephalitis who had been admitted to five hospitals across China between January 2013 and December 2014. The hospitals were the Affiliated Second Hospital of Wenzhou Medical University (Wenzhou, China), the First People's Hospital of Yongkang (Jinhua, China), the Affiliated Children's Hospital of Zhejiang University Medical College (Hangzhou, China), Hainan Provincial People's Hospital (Haikou, China), and Zouping People's Hospital (Binzhou City, Shandong Province, China). This study was approved by the Institutional Ethics Committee of the Affiliated Second Hospital of Wenzhou Medical University (approval number: 1-2016-12). The inclusion criteria were as follows: (1) confirmation of HFMD with encephalitis based on the diagnostic criteria of the Guidelines for the Diagnosis and Treatment of HFMD (2012 edition, China), including scattered blisters on the oral mucosa, maculopapules, and vesicles on the hands, feet, and buttocks; (2) patients aged >1 year (since* S. baicalensis* cannot be applied to children aged <1 year); (3) nervous system (NS) involvement; (4) infection by EV71; (5) onset within 48 hours; (6) no antiviral drug use prior to the disease course; and (7) normal functioning of major organs (the heart, liver, and kidney). Indicators of NS involvement included a poor spirit, lethargy, being easily frightened, delirium, headache and vomiting, limb jitters, myoclonus, nystagmus, ataxia, an eyeball movement disorder, weakness, acute flaccid paralysis, convulsions, signs of irritation, and weakening or disappearance of tendon reflexes. In addition, abnormal cerebrospinal fluid (CSF), electroencephalograph (EEG), or magnetic resonance imaging (MRI) findings of the brain were also considered as indicators of NS involvement. The exclusion criteria were as follows: (1) mild HFMD; (2) patients aged <1 year; (3) prior use of antiviral drugs, including interferon-*α*, ribavirin, and* S. baicalensis* injection, or other oral traditional medications; (4) use of glucocorticoids and/or intravenous immunoglobulin (IVIG) prior to and/or within 3 days after hospitalization; and (5) the development of coexisting severe diseases, including cardiac, respiratory, liver, and renal dysfunctions, or severe malnutrition, on the first day of hospitalization. Since only intravenous injections of ribavirin or* S. baicalensis* are recommended according to the guidelines, the cases were divided into the ribavirin and* S. baicalensis* groups.* S. baicalensis *was purified and made into a Tanreqing injection. All patients accepted one of the antiviral injection treatments on the first hospitalization day. Patients in the* S. baicalensis* group received a 0.3–0.5 mL/kg injection of* S. baicalensis *once a day, diluted in a 100–200 mL 5% glucose injection solution or 0.9% sodium chloride injection solution for intravenous infusion at a speed of 1.5–3.0 mL/min. Patients in the ribavirin group received a 10 mg/kg injection of ribavirin once a day. In addition, other medications, including mannitol and neurotrophic drugs, were regularly administered for symptomatic and supportive purposes. Data retrieved from the medical records were the demographic and clinical characteristics of the patients, the EV71 viral load, outcome of medication, prognosis, and safety of the treatments. Patient records and information in the database were anonymized and deidentified prior to analysis.

The following parameters and symptoms were analyzed as indicators of drug efficacy: (1) body temperature was initially measured once every 4 hours and, following its return to normal for 24 hours, was measured once every 8 hours; (2) the hands, feet, buttocks, and other parts of the skin were checked daily for signs of crusting and the presence or absence of rashes, as well as the appearance of new rashes; (3) oral ulcers and/or vesicles were checked daily for lesion crusting and to determine whether they had cleared; (4) attenuation of NS involvement was recorded and investigated; (5) pathogenic detection of throat swabs was performed on days 0, 3, and 7 of the treatment course and on days 0 and 7 using CSF samples, which were then analyzed by the Centers for Disease Control for EV71 nucleic acid using reverse transcription-polymerase chain reaction (RT-PCR). Using fluorescence quantitative PCR to detect viral load, the total RNA in the blood sample was extracted and the VP1 gene was amplified by EV71 specific primers (EV71-1/F: 5′-ATAATAGCAYTRGCGGCAGCCCA-3′, EV71-2/R: 5′-AGAGGGAGRTCTATCTCYCC-3′). The expected amplified fragment length was 379 bp. With GAPDH as the reference, the upstream primer/F was 5′-CCATCACCATCTTCCAGGAG-3′, downstream primer/R was 5′-CCTGCTCACCACCTTCTTG-3′, and product length was 452 bp.

**Figure 1:**
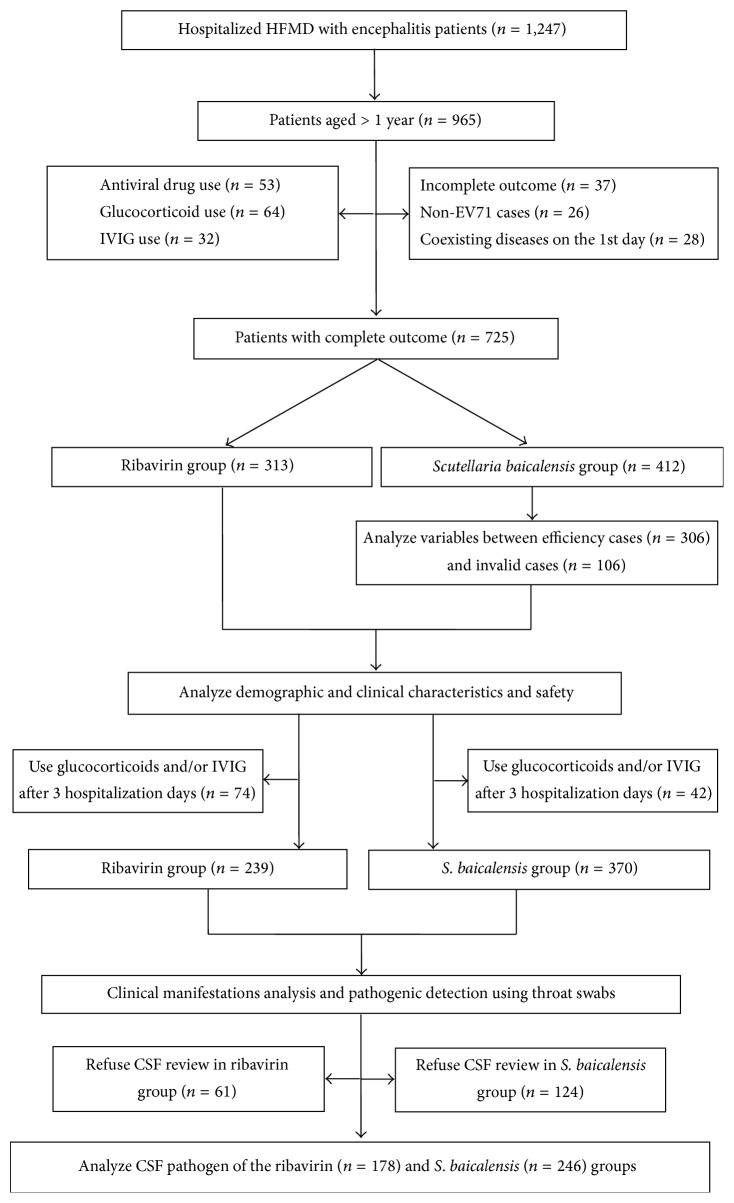
Flow chart of patients included in the investigation. HFMD: hand, foot, and mouth disease; EV71: enterovirus 71; IVIG: intravenous immunoglobulin; CSF: cerebrospinal fluid.

The therapeutic effects were assessed in both groups using the established criteria of ratings for effectiveness and invalid observations. An “efficiency” rating was given when the temperature of the patient returned to normal; the hand, foot, and hip rashes were attenuated; the oral ulcers and/or vesicles had cleared; and the NS involvement was improved within 3 days. Conversely, an “invalid” rating was given when the fever persisted; the rashes and oral ulcers/vesicles showed no signs of reduction or were even increased; and the patient showed worsened NS involvement after 3 days of treatment (Figure 1).

## 3. Analysis

Data processing and statistical analyses were performed using the Statistical Package for the Social Sciences (SPSS) version 17.0 software. The results of each group were compared using the *t*-test or Chi-square test (including Fisher's exact test) and with logistic regression analysis.

## 4. Results

### 4.1. Demographic and Clinical Characteristics

A total of 725 patients were enrolled in the present study, including 313 patients in the ribavirin group (98 patients from the Affiliated Second Hospital of Wenzhou Medical University, 41 from the First People's Hospital of Yongkang, 76 from the Affiliated Children's Hospital of Zhejiang University Medical College, 52 from the Hainan Provincial People's Hospital, and 46 from Zouping People's Hospital) and 412 patients in the* S. baicalensis* group (122 patients from the Affiliated Second Hospital of Wenzhou Medical University, 56 from the First People's Hospital of Yongkang, 108 from the Affiliated Children's Hospital of Zhejiang University Medical College, 74 from the Hainan Provincial People's Hospital, and 52 from Zouping People's Hospital).

The majority of the included patients were male (59.4%). The mean age of the patients was 20.6 ± 5.9 months; 54.9% of the patients were from urban areas; 54.9% of the patients had received medication prior to hospitalization; and 12.7% had a history of HFMD. All patients exhibited symptoms, including fever, skin rashes, oral lesions, and NS involvement. In addition, the mean fasting blood glucose (FBG) level of the patients was 6.5 ± 2.3 mmol/L, the mean peripheral white blood cell (PWBC) count was (10.8 ± 2.8) × 10^9^/L, and the mean CSF-WBC count was (179.3 ± 129.6) × 10^6^/L. Furthermore, 63.2% and 27.7% of the patients showed abnormal EEG and brain MRI findings, respectively. No significant differences were observed in the occurrence of symptoms, including fever, rashes, oral lesions, and NS involvement, as well as the sex, age, location of residence, FBG level, PWBC and CSF-WBC counts, and abnormal EEG and brain MRI findings, between the two groups (all *P* > 0.05; Table 1).

### 4.2. Clinical Manifestations and Pathogenic Detection

**Figure 2:**
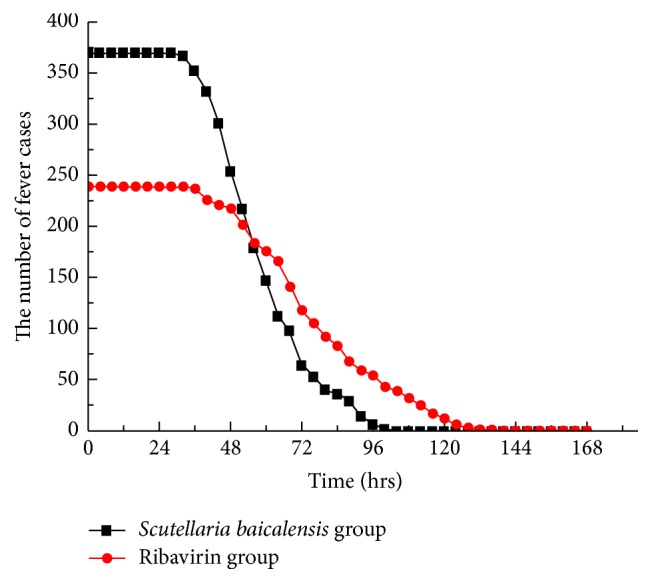
Number of fever cases in both groups. The total number of patients that presented with a fever in both groups was 609, including 370 in the* Scutellaria baicalensis* group and 239 in the ribavirin group. The interval between each time point was 4 hours. Temperatures above 37.5°C were considered to indicate a fever and those maintained below 37.5°C for >24 hours were considered normal.

**Figure 3 fig3:**
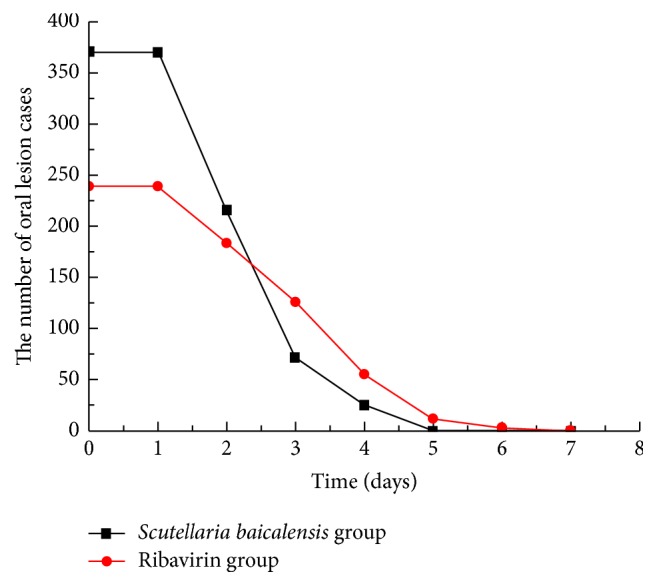
Number of oral lesions in both groups. Disappearance of oral ulcers and/or vesicles was considered a return to normal.

**Figure 4 fig4:**
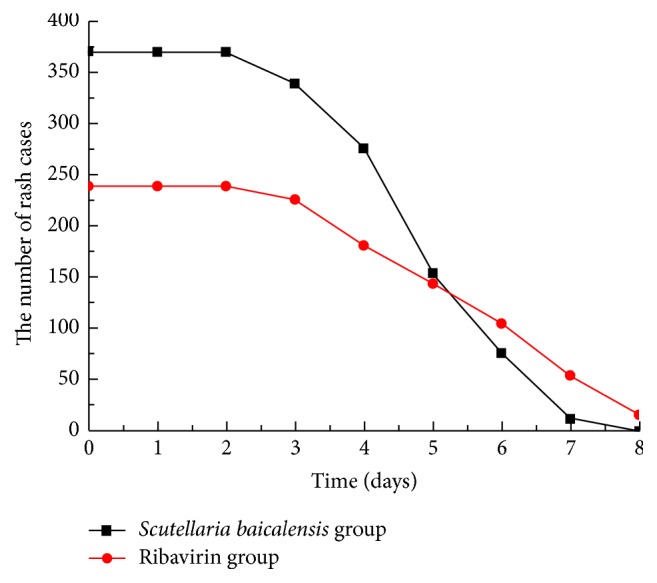
Number of rash cases in both groups. The disappearance of rashes was considered a return to normal condition.

**Figure 5:**
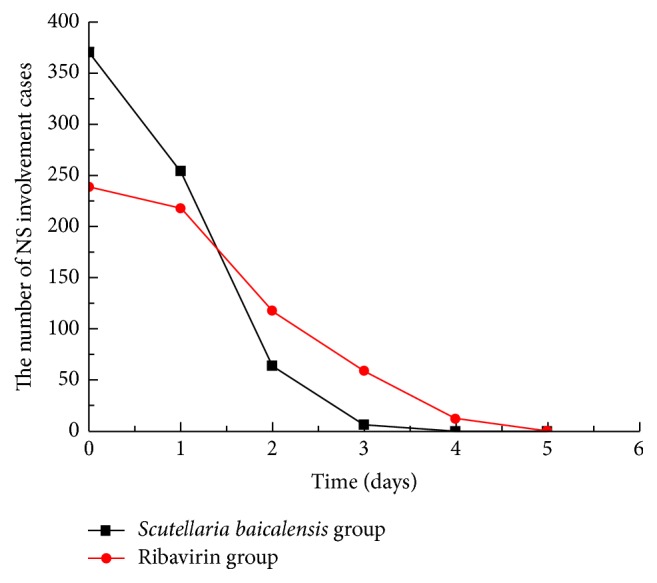
Number of patients with NS involvement in both groups. The disappearance of NS-associated syndromes was considered a return to normal condition. NS: nervous system.

A number of patients with NS involvement showed progression in the course of the disease, including the development of brainstem encephalitis or neurogenic pulmonary edema; for such patients, the use of corticosteroids and high-dose IVIG was deemed necessary to reverse the progression of the disease. The use of these therapies has previously been shown to promote a rapid return to normal body temperature and to attenuate NS involvement [6, 7]. Therefore, patients where disease progression occurred within 3 days following hospitalization were excluded from this study, since the use of these drugs would confound the effects of antiviral therapy. However, those patients in whom disease progression occurred after 3 days of hospitalization remained in the study but were classified as treatment invalid cases and were not included in the analysis of clinical characteristics. These invalid cases constituted 116 patients (16% of all cases), including 42 patients in the* S. baicalensis* group and 74 patients in the ribavirin group. Using only the data of the patients who did not receive corticosteroids or IVIG treatment (609 patients, including 370 in the* S. baicalensis* group and 239 in the ribavirin group), the median duration of fever in the* S. baicalensis* group was significantly shorter compared with that in the ribavirin group (56.9 ± 16.1 and 78.3 ± 23.7 hours, resp.; 95% confidence intervals [CI] = 55.25–58.55 and 75.28–81.32 hours, resp.; *t* = 13.2847, *P* < 0.0001). In addition, the median duration of oral ulcer and/or vesicle persistence was significantly shorter in the* S. baicalensis* group compared with the ribavirin group (2.85 ± 0.89 and 3.59 ± 1.2 days, resp.; 95% CI = 2.76–2.94 and 3.44–3.74 days, resp.; *t* = 8.6409, *P* = 0.0000). Furthermore, there was a significant difference in the median duration of rashes between the* S. baicalensis* and the ribavirin groups (5.32 ± 1.27 and 5.97 ± 1.59 days, resp.; 95% CI = 5.19–5.45 and 5.77–6.17 days, resp.; *t* = 5.5246, *P* = 0.0000). The median time to attenuation of NS involvement in the* S. baicalensis* group was significantly shorter compared with the ribavirin group (2.88 ± 0.72 and 3.69 ± 0.98 days, resp.; 95% CI = 2.81–2.95 and 3.57–3.81 days, resp.; *t* = 11.7359, *P* = 0.0000) (Figures 2 [Fig fig3] [Fig fig4]–5).

### 4.3. Viral Load Detection of EV71

The enteroviral nucleic acid was detected thrice for throat swab samples and twice for CSF samples from the two groups using RT-PCR (for quantitative detection), and the mean viral loads were compared. The actual CSF viral load was detected for only 424 of the 609 patients who did not receive corticosteroids or IVIG treatment, as some patients refused the CSF analysis (124 and 61 patients in the* S. baicalensis* and ribavirin groups, resp.). There were no significant differences between the groups in terms of the throat swab viral loads (*t* = 1.1251, *P* = 0.2610) and CSF viral loads (*t* = 0.4385, *P* = 0.6612) at the first RT-PCR detection of EV71. Conversely, there were significant differences between the groups in the throat swab viral loads at the second (*t* = 40.8935, *P* = 0.0000) and third (*t* = 35.9431, *P* = 0.0000) detection and the CSF viral loads at the second detection (*t* = 27.4736, *P* = 0.0000) (Table 2).

### 4.4. Prognosis

The efficacy of* S. baicalensis* treatment was analyzed based on the data, and patients who used glucocorticoids or IVIG after 3 days of hospitalization were classed as invalid. In total, there were 306 efficiency cases and 106 invalid cases in the* S. baicalensis* group (*n* = 412) and 121 efficiency cases and 192 invalid cases in the ribavirin group (*n* = 313), which was significantly different (*χ* ^2^ = 93.1901, *P* = 0.0000).

The occurrence of adverse events (AEs) and serious AEs (SAEs) was evaluated for both groups. The main SAEs were elevated levels of alanine aminotransferase (ALT) and creatine kinase-MB (CK-MB), brainstem encephalitis, and neurogenic pulmonary edema. There were statistically significant differences between the two groups in terms of the occurrence of SAEs (all *P* < 0.05). These SAEs were not considered significantly drug-related but were considered as disease progression. There were no cases of gastroenteritis, kidney damage, or hematological disorders in both groups. Skin allergies only occurred in the* S. baicalensis* group, which was potentially associated with the AEs caused by* S. baicalensis* (Table 3).

Although the efficacy rate for the* S. baicalensis* group was 74.3%, 106 cases showed disease progression. Therefore, the present study analyzed the variables between the efficiency and invalid cases. Notably, there were significant differences between the efficiency and invalid cases in the age of the patients; the occurrence of a fever of >39°C over 3 days; the symptoms of limb jitters, nystagmus, ataxia, and acute flaccid paralysis; the FBG level; and PWBC and CSF-WBC counts (all *P* < 0.05). However, there was no significant difference between the efficiency and invalid cases in the sex of the patients, the symptoms of a poor spirit, headache and vomiting, convulsions, signs of irritation, and EEG and MRI abnormalities (*P* > 0.05; Table 4). A logistic regression analysis was performed to compare the variables and showed that the *R* ^2^ value of the model was 0.925. The correct rate of validity prediction was 97.6%. The analysis also demonstrated that the age, FBG level, and PWBC counts were statistically significant (*P* = 0.001, 0.001, and 0.003, resp.), indicating the effectiveness of these three variables on treatment effects. The odds ratios (95% CIs) for these three variables were 1.43 (1.165–1.755), 0.551 (0.385–0.787), and 0.689 (0.537–0.884), respectively (Table 5).

## 5. Discussion

In China, the interest in HFMD has increased in recent years, even though HFMD is not a new infectious disease. This is a result of the HFMD outbreak in China from March to May 2008 [8] and the fact that the disease has shown seasonal epidemic patterns in China in recent years. On May 2, 2008, the Chinese Ministry of Health included HFMD in the class C infectious disease management category [9]. It has been confirmed that almost all severe cases of HFMD with encephalitis are caused by the EV71 virus [7]. Although the majority of the severe cases can be cured, there are some examples of severe cases where the disease has progressed into deadly complications, including brainstem encephalitis and neurogenic pulmonary edema [7, 10].

At present, there is no effective antiviral therapy for the EV71 virus; although ribavirin is recommended as an antiviral medication, the actual clinical efficacy of ribavirin is poor [4]. Some traditional Chinese medications have been reported to exert antiviral effects, including Gan-Lu-Siao-Du-yin [11] and Jinzhen oral liquid [12], which have been assessed for their ability to treat HFMD. However, all of the traditional Chinese medications are oral preparations, and the biological mechanisms underlying their effects on the clinical course of HFMD are unknown.* S. baicalensis* injection is the only herbal medication extract that has been recommended by the Guidelines for the Diagnosis and Treatment of HFMD (2012 edition, China). In previous studies,* S. baicalensis *demonstrated effective anti-inflammatory and antiviral activities in the treatment of cancer and viral infectious diseases [13–15]. In addition, previous studies have suggested that these effects of* S. baicalensis* were associated with the regulation of multiple molecular pathways, including reactive oxygen species, Ca^2+^, nuclear factor-*κ*B, and tumor necrosis factor-related apoptosis-inducing ligand [16–19]. Previous research published in Chinese domestic medical journals indicated that* S. baicalensis *induced EV71 virus infected mice produced IFN-*α*, which had an obvious promoting effect on the phagocytic and proliferation functions of B and T lymphocytes and peritoneal macrophages. IFN-*α* also released the inhibition of Th2 cells secreting IL-4 and promoted the secretion of IFN-*γ*, which indicates the potential anti-EV71 mechanism of* S. baicalensis*. There have been a number of single center studies that have investigated the efficacy of applying* S. baicalensis* injection to the treatment of HFMD, and the majority of these have been published in Chinese domestic medical journals. However, no multicenter analysis has yet demonstrated the antiviral effects of* S. baicalensis* injection in the treatment of EV71 infections.

As the* S. baicalensis* injection is already in clinical use, we retrospectively analyzed data from five hospitals distributed across China. The parameters evaluated included the duration of fever, persistence of oral ulcers and/or vesicles, the time taken for skin rashes to subside, and NS involvement. Specifically, we investigated the reduction in the number of daily fever episodes and demonstrated that the* S. baicalensis* group exhibited a more rapid reduction in temperature compared with the ribavirin group. Furthermore, the* S. baicalensis* group showed a more rapid improvement in the time required to resolve the incidence of oral ulcers and/or vesicles, as well as clear skin rashes and attenuate NS involvement, as compared with the ribavirin group. Although there were patients in both groups (42 and 74 patients in the* S. baicalensis* and ribavirin groups, resp.) who presented with NS involvement associated with further disease progression, the proportion in the* S. baicalensis* group was significantly lower compared with the ribavirin group. In addition, the EV71 viral loads of throat swabs and CSF samples from both groups showed that* S. baicalensis* exerted more potent antiviral activity compared with ribavirin. Overall, the differences in improvements to severe HFMD symptoms, including fever, the presence of oral lesions and skin rashes, NS involvement, and high EV71 viral load, between the two groups were small; however, they suggested that* S. baicalensis* was more effective than ribavirin.

Although the results of the present study suggested that* S. baicalensis* was superior to ribavirin for the treatment of HFMD, the different states of the patients might have contributed to the observed differences in the therapeutic effects. The age of the patients likely affected the impact of the treatments, whereas the sex distributions were not significantly different between the efficiency and invalid cases in the* S. baicalensis* group. The comparison of the data based on age suggested that the treatments were more effective in older patients, which may be attributable to the presence of a more highly developed immune system in these patients [20]. A fever of >39°C over 3 days has previously been confirmed as an important indicator of severe HFMD cases; a persistent high fever for >3 days has been shown to predict a poor prognosis [3, 21]. NS involvement is the predominant manifestation of severe HFMD, and the degree of involvement was shown to predict the patient prognosis [3, 22]. In the present study, NS involvement variables, including limb jitters, nystagmus, ataxia, and acute flaccid paralysis, were predictors of treatment failure in severe cases, whereas the variables of a poor spirit, headache and vomiting, convulsions, and signs of irritation, were not significantly different between the efficiency and invalid cases of the* S. baicalensis* group, which is consistent with previous studies [5, 23, 24]. In addition, the FBG level and PWBC count were shown to influence the effectiveness of* S. baicalensis *injection, and both have been reported to be significantly increased in patients with severe HFMD [25]. Conversely, CSF-WBC counts and EEG and brain MRI abnormalities did not appear to influence the efficacy of the treatment. A logistic regression analysis indicated that older age was associated with increased therapeutic effectiveness, that increased FBG levels and PWBC counts could reduce the treatment effectiveness, and that NS involvement and EEG and brain MRI abnormalities were not indicators of the efficacy of* S. baicalensis* for the treatment of HFMD.

The incidence of AEs, including a skin allergy, was significantly different between the* S. baicalensis* and ribavirin groups, which suggested that skin allergies are a side effect of* S. baicalensis* treatment, since no skin allergy was observed in the ribavirin group. The occurrence of SAEs, including neurogenic pulmonary edema and brainstem encephalitis, as well as elevated levels of CK-MB and ALT, was significantly different between the groups and was more common in patients with disease progression associated with NS involvement, which is also regarded as a severe clinical manifestation of HFMD [26]. In addition, the results suggested that treatment with* S. baicalensis* prevented the progression of the disease and the occurrence of SAEs, thus suggesting that* S. baicalensis* was relatively safe.

## 6. Conclusions

This multicenter analysis demonstrated that* S. baicalensis* was suitable for the treatment of severe HFMD, as evidenced by its rapid attenuation of fever, promotion of oral lesion and rash subsidence, and improvement of NS involvement in patients with HFMD. In addition,* S. baicalensis* was determined to be safe for topical application.

## Figures and Tables

**Table 1:** Demographic and clinical characteristics of cases.

Variable	*Scutellaria baicalensis* group	Ribavirin group	*χ* ^2^/*t*-test (*P* value)
Sex			
Male	238 (57.8)	193 (61.7)	1.1190 (0.2901)
Female	174 (42.2)	120 (38.3)	
Age (months)	20.8 ± 5.9	20.1 ± 6.2	1.5479 (0.1221)
Location of residence			
Urban	220 (53.4)	178 (56.7)	0.8655 (0.3522)
Rural	192 (46.6)	135 (43.3)	
Medication use before hospitalization			
Yes	274 (66.5)	200 (63.9)	0.5341 (0.4649)
No	138 (33.5)	113 (36.1)	
Previous history of HFMD			
Yes	56 (13.6)	36 (11.5)	0.4207 (0.5166)
No	356 (86.4)	277 (88.5)	0.7017 (0.4022)
Symptoms			
Fever	412 (100.0)	313 (100.0)	— (1.0000)
Skin rash	412 (100.0)	313 (100.0)	— (1.0000)
Oral lesion	412 (100.0)	313 (100.0)	— (1.0000)
NS involvement	412 (100.0)	313 (100.0)	— (1.0000)
FBG level (mmol/L)	6.6 ± 2.2	6.4 ± 2.4	1.1656 (0.2442)
PWBC count (×10^9^/L)	10.6 ± 2.7	10.8 ± 3.1	0.9263 (0.3546)
CSF-WBC count (×10^6^/L)	183.9 ± 128.3	174.5 ± 130.2	0.9709 (0.3319)
EEG abnormality	262 (63.6)	196 (62.6)	0.0723 (0.7880)
MRI abnormality	112 (27.2)	89 (28.4)	0.1387 (0.7096)

A total of 725 patients with HFMD were enrolled in the present study, including 412 patients in the *Scutellaria baicalensis* group and 313 in the ribavirin group. Data are presented as *n* (%) or the mean ± standard deviation. HFMD: hand, foot, and mouth disease; NS: nervous system; FBG: fasting blood glucose; PWBC: peripheral white blood cells; CSF: cerebrospinal fluid; EEG: electroencephalograph; MRI: magnetic resonance imaging. The data for the clinical manifestations were compared using Fisher's exact test.

**Table 2:** Enterovirus 71 viral loads in the throat swab and CSF samples from both groups.

Sample	*Scutellaria baicalensis* group	Ribavirin group	*t*-test (*P* value)
Throat swab			
First (×10^6^)	6.81 ± 3.45	6.47 ± 3.92	1.1251 (0.2610)
Second (×10^6^)	1.26 ± 0.52	4.48 ± 1.37	40.8935 (0.0000)
Third (×10^3^)	6.4 ± 4.7	73.6 ± 35.5	35.9431 (0.0000)
CSF			
First (×10^6^)	6.69 ± 4.01	6.52 ± 3.84	0.4385 (0.6612)
Second (×10^3^)	2.7 ± 1.6	58.3 ± 31.7	27.4736 (0.0000)

The viral loads of the throat swab samples from 609 patients, including 370 patients in the *Scutellaria baicalensis* group and 239 patients in the ribavirin group. Viral loads were determined for the CSF samples from 424 patients, including 246 patients in the *S. baicalensis* group and 178 patients in the ribavirin group. CSF: cerebrospinal fluid.

**Table 3:** Adverse events (AEs) and severe AEs observed in both groups.

Variable, number	*Scutellaria baicalensis* group	Ribavirin group	*χ* ^2^/Fisher (*P* value)
Skin allergy	12	0	— (0.0017)
Gastroenteritis	0	0	— (—)
Neurogenic pulmonary edema	2	8	4.1867 (0.0407)
Brainstem encephalitis	9	17	5.4232 (0.0199)
Elevated CK-MB	8	19	8.4558 (0.0036)
Elevated ALT	16	27	7.1710 (0.0074)
Kidney damage	0	0	— (—)
Hematological disorder	0	0	— (—)

Skin allergy data were compared using Fisher's exact test. There were no cases of gastroenteritis, kidney damage, or hematological disorders in either group. CK-MB: creatine kinase-MB; ALT: alanine aminotransferase.

**Table 4:** Comparison of efficiency and invalid cases in the *Scutellaria baicalensis* group.

Variable	Efficiency (*n* = 306)	Invalid (*n* = 106)	*χ* ^2^/*t*-test (*P* value)
Sex			
Male	165 (53.9)	58 (54.7)	0.0201 (0.8874)
Female	141 (46.1)	48 (45.3)	
Age (months)	22.3 ± 6.0	16.7 ± 3.8	6.4962 (0.0000)
Fever >39°C over 3 days	26 (8.5)	92 (86.8)	236.1409 (0.0000)
NS involvement			
Poor spirit	207 (67.6)	76 (71.7)	0.6007 (0.4383)
Headache and vomiting	196 (64.1)	72 (67.9)	0.5192 (0.4712)
Limb jitters	12 (3.9)	46 (43.4)	101.4215 (0.0000)
Nystagmus	0 (0.0)	16 (15.1)	— (0.0000)
Ataxia	1 (0.3)	8 (7.5)	15.9781 (0.0001)
Acute flaccid paralysis	6 (1.96)	17 (16.0)	29.5981 (0.0000)
Convulsion	45 (14.7)	22 (20.8)	2.1153 (0.1458)
Irritation sign	152 (49.7)	61 (57.5)	1.9547 (0.1621)
FBG level (mmol/L)	6.0 ± 2.0	8.5 ± 1.9	7.3916 (0.0000)
PWBC count (×10^9^/L)	9.9 ± 2.2	12.6 ± 3.0	10.0999 (0.0000)
CSF-WBC count (×10^6^/L)	185.6 ± 132.9	179.1 ± 115.4	1.5829 (0.1142)
EEG abnormality	194 (63.4)	68 (64.2)	0.2566 (0.6125)
MRI abnormality	80 (26.1)	32 (30.2)	0.6507 (0.4199)

A fever of >39°C over 3 days referred to 39°C at least once a day and persisted for more than 3 days, with no downward trend. Nystagmus data were compared using Fisher's exact test. NS: nervous system; FBG: fasting blood glucose; PWBC: peripheral white blood cells; CSF-WBC: cerebrospinal fluid-white blood cells; EEG: electroencephalograph; MRI: magnetic resonance imaging.

**Table 5:** Logistic analysis of the efficiency and invalid cases in the *Scutellaria baicalensis* group.

Variable	Efficiency	Invalid	*P* value	ORuadj (95% CI)	ORadj (95% CI)
Age (months)	22.3 ± 6.0	16.7 ± 3.8	0.001	1.356 (1.256–1.465)	1.43 (1.165–1.755)
FBG level (mmol/L)	6.0 ± 2.0	8.5 ± 1.9	0.001	0.523 (0.449–0.609)	0.551 (0.385–0.787)
PWBC count (×10^9^/L)	9.9 ± 2.2	12.6 ± 3.0	0.003	0.673 (0.611–0.741)	0.689 (0.537–0.884)

There were 412 patients in the *Scutellaria baicalensis* group, of whom 306 were efficiency cases and 106 were invalid cases. FBG: fasting blood glucose; PWBC: peripheral white blood cells; 95% CIs: 95% confidence intervals; OR: odds ratio.
